# The role of selenoproteins in neurodevelopment and neurological function: Implications in autism spectrum disorder

**DOI:** 10.3389/fnmol.2023.1130922

**Published:** 2023-03-09

**Authors:** Supriya Behl, Sunil Mehta, Mukesh K. Pandey

**Affiliations:** ^1^Department of Pediatric and Adolescent Medicine, Mayo Clinic, Rochester, MN, United States; ^2^Center for Childhood Cancer Research, Children’s Hospital of Philadelphia, Philadelphia, PA, United States; ^3^Department of Radiology, Mayo Clinic, Rochester, MN, United States

**Keywords:** metal micronutrients, selenium, autism spectrum disorder, brain function, brain development

## Abstract

Selenium and selenoproteins play a role in many biological functions, particularly in brain development and function. This review outlines the role of each class of selenoprotein in human brain function. Most selenoproteins play a large antioxidant role within the brain. Autism spectrum disorder (ASD) has been shown to correlate with increased oxidative stress, and the presumption of selenoproteins as key players in ASD etiology are discussed. Further, current literature surrounding selenium in ASD and selenium supplementation studies are reviewed. Finally, perspectives are given for future directions of selenoprotein research in ASD.

## Introduction

**Figure 1:**
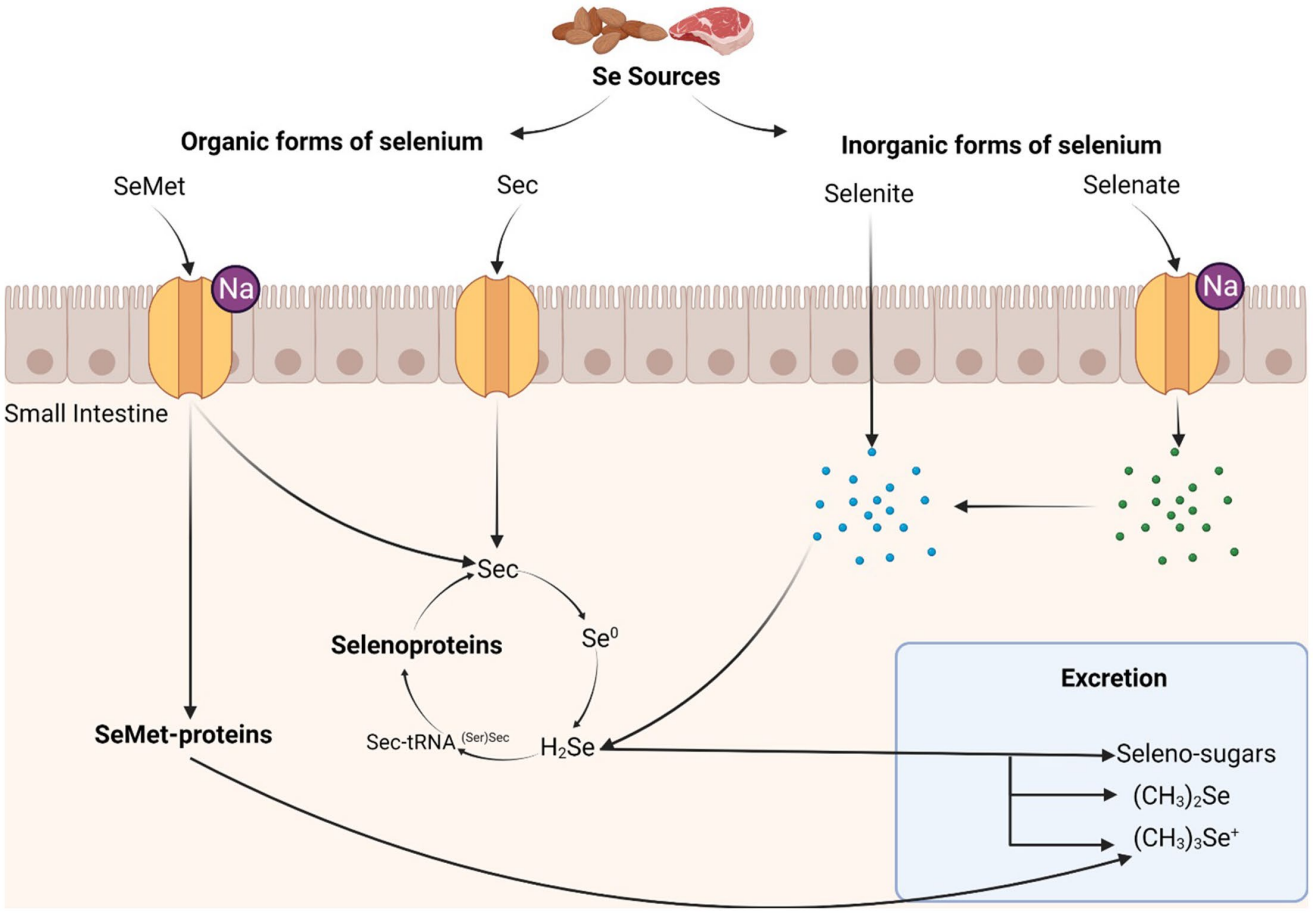
Metabolism of selenium in the human body. High sources of selenium include most red meats and some forms of nuts. Selenomethionine (SeMet) is transported across the brush-border membrane of the small intestine through sodium-mediated channels, while selenocysteine (Sec) is transported using mechanisms similar to cysteine. Organic forms of selenium are translated into selenomethionine or selenocysteine-containing proteins. Selenite is transported across the brush-border membrane by passive diffusion, while selenate uses a sodium-mediated channel. Inorganic forms of selenium are either incorporated into selenoproteins or excreted. Selenite, unlike other forms of selenium, is transported through passive diffusion, though this is much slower than transport of other selenium forms. Selenate is converted into selenite before entering the selenoprotein synthesis cycle.

Selenium (Se) is a human micronutrient and is critical for many biological functions. Selenium is ingested from solid food sources and absorbed as selenomethionine and selenocysteine (Ha et al., 2019). The US Food and Nutrition Board at the Institute of Medicine of the National Academies recommends a daily dietary reference intake of 55 mcg in both men and women aged 14 years and older, with a slightly higher intake recommendation for those who are pregnant or lactating (Institute of Medicine, 2000). Once absorbed in the small intestine, selenium is incorporated into one of two Se-containing proteins *in vivo*: (a) selenomethionine-containing proteins, which are nearly identical to their methionine-containing counterparts (Schrauzer, 2000); and (b) selenocysteine (Sec)-containing proteins, which are specialized proteins essential to human function (Figure 1). Sec-containing proteins are most biologically relevant and are commonly known as selenoproteins (Minich, 2022).

Approximately 80% of selenium is found in association with selenoproteins (Minich, 2022). To date, there are 25 known selenoproteins (Kryukov et al., 2003). There are three major classes of selenoproteins (Pitts et al., 2014). First, glutathione peroxidases function to protect from oxidative damage by reducing hydrogen peroxide and peroxide radicals. There are eight known glutathione peroxidases, but only five (GPX1, GPX2, GPX3, GPX4, and GPX6) have been associated with selenocysteine in their active sites. Thioredoxin reductases (Trx1, Trx2, and Trx3) reduce disulfides to oxidize thiol-dependent peroxidases, thereby exhibiting antioxidant activity as well (Lu and Holmgren, 2014). Thioredoxin reductases also play a role in cell growth and p53 activity (Mustacich and Powis, 2000). Deiodinases (DIO1, DIO2, and DIO3) primarily assist in regulating plasma thyroid hormone (T_3_) levels, and conversion of T_3_ to T_4_ (Luongo et al., 2019). The remainder of selenoproteins are named with the prefix SELENO-and conduct a variety of functions, such as reduction of lipid hydroperoxides and regulation of protein folding and endoplastic reticulum (ER) stress (Zhang and Song, 2021). With the exception of deiodinases, all selenoproteins have some method of antioxidant activity. A summary of selenoproteins and their functions are included in Table 1.

**Table 1:** Twenty-Five selenoproteins along with expression in brain and blood and major functions in brain.

Selenoprotein	Expressed in brain (Zhang et al., 2008)	Expressed in blood (Uhlen et al., 2010)	Major findings/function in brain	Source
GPX1	Yes	Yes	Clearance of H_2_O_2_	36, 39
			GPX1 variant potentially associated with ASD in one case	
GPX2	Yes-Low	Yes	Insufficient literature	
GPX3	Yes-Low	Yes	Insufficient literature	
GPX4	Yes-High	Yes	Protection from lipid peroxidation and ferroptosis	33, 34
GPX6	No	No	Not expressed in brain	
Trx1	Yes	Yes	Inhibits intracellular ROS levels and DNA fragmentation	49
Trx2	Yes	Yes	Inhibits intracellular ROS levels	45
Trx3	Yes	No	Insufficient literature	
DIO1	Yes-Low	No	Activates thyroid hormones T3 and T4	50
DIO2	Yes	No	Activates thyroid hormones T3 and T4	50
			Regulation of ER homeostasis	
DIO3	Yes-Low	No	Inactivates thyroid hormones T3 and T4	50
SELENOH	Yes	No	Insufficient literature; antioxidant properties	10
SELENOK	Yes	No	Regulation of ER homeostasis	10
			May play a role in neuronal synaptic transmission	
SELENOM	Yes	Yes	Regulation of ER homeostasis	10
SELENON	Yes	No	Regulation of ER homeostasis	10
			Regulation of Ca^2+^ flux and protein folding	
SELENOO	Yes	No	Insufficient literature; antioxidant properties	10
SELENOP	Yes-High	Yes	Transportation of selenoproteins to brain	18, 19
SELENOR	Yes	Unknown	Antioxidant function within hippocampus	10, 69
SELENOS	Yes	No	Regulation of ER homeostasis	10
SELENOT	Yes	No	Redox homeostasis during brain ontogenesis	71
			Regulation of ER homeostasis	
SELENOV	Yes-Low	No	Insufficient literature; antioxidant properties	10
SELENOW	Yes-High	No	Interacts with d-amino acid oxidase downstream of d-serine signaling to prevent oxidative stress	10, 74, 75
SELENOI/EPT1	Yes	No	Not directly involved in antioxidant activity, but the products of its function (i.e., phosphatidylethanolamine) play a role in defending against oxidative stress	83
SELENOF	Yes-High	Yes	Regulation of ER homeostasis	10
			Regulation of Ca^2+^ flux and protein folding	
SEPHS2	Yes	Yes	Selenocysteine (Sec) biosynthesis	10

Selenoprotein activity is essential to many biological processes, such as fetal development (Hogan and Perkins, 2022), hormone regulation, and reproduction (Kurokawa and Berry, 2013). Selenium deficiency during pregnancy leads to reduced placental weight and reduced fetal blood glucose levels in mice (Hofstee et al., 2019), likely due to deficiencies in SELENOP and SELENON. In addition, methylation at Se-dependent CpG sites during pregnancy is associated with poorer muscle tone in newborns (Tian et al., 2020). Selenium is highly present in the testes as well, and selenium deficiency adversely impacts testes development and spermatozoa production (Behne et al., 1996). Several selenoproteins (GPX1, GPX2, GPX3, GPX4, SELENOM, SELENOP, SELENOS, and SELENBP1) are expressed in the intestine, and they may play a role in mitigating excessive immune responses that may lead to inflammatory bowel diseases (Speckmann and Steinbrenner, 2014).

Most known selenoproteins are expressed in the neurons of adult mouse brains (Zhang et al., 2008) and in human brains (Zhang and Song, 2021). Within the brain, there is the most selenoprotein expression in the olfactory bulb, cerebral cortex, hippocampus, and cerebellar cortex (Zhang et al., 2008), and selenium is transported to these areas by SELENOP (Hill et al., 2003; Schomburg et al., 2003). When selenoprotein formation is malfunctioning, studies have shown cerebellar hypoplasia and atrophy and cerebral atrophy (Agamy et al., 2010; Wirth et al., 2014), indicating the necessity of selenoproteins during brain development. Therefore, deficiencies in selenium and/or selenoproteins poses some threat for neurological conditions (Schweizer et al., 2021).

Autism spectrum disorder (ASD) affects 1 in 44 children in the United States (Maenner et al., 2021), and is characterized by repetitive behaviors and issues with social interaction (Mughal et al., 2022). A recent study from our group demonstrated lower serum and nail selenium levels in boys aged 24 to 47 months with ASD compared to sex-matched controls (Mehta et al., 2021). In addition, there was a negative correlation between Autism Diagnostic Observation Schedule (ADOS) scores and selenium levels. The purpose of this review is to examine the role of selenium and selenoproteins in the human brain, and how this may be implicated in ASD etiology. To our knowledge, there has not been a quantitative study of Selenoproteins in people with ASD.

## Selenium and selenoproteins in brain development and function

### Role of selenium in the brain

In a study examining the proportions of total body selenium in individual organs, it was found that 2.4% of total body selenium is found in the brain (Zachara et al., 2001). When selenium is deprived from the diets of four generations of mice, an increased brain uptake of selenium was identified (Kuhbacher et al., 2009), suggesting the strong need for selenium homeostasis in the brain. When this homeostasis is disturbed, an evident difference is noticed in neurological function and ability. For example, in a cohort of over 1,000 Italian participants, plasma selenium levels were inversely related to neuro-motor performance outcomes, such as finger taps and heel-tibia cycles (Shahar et al., 2010). A systematic review and meta-analysis also identified an inverse correlation between selenium intake and depressive symptoms (Sajjadi et al., 2022). When the gene encoding the Sec tRNA was deleted in mice, significant neurodegeneration was identified by postnatal day 6 (P6) (Wirth et al., 2010). Taken together, selenium likely plays a critical role in neurodevelopment and neurological function.

### Glutathione peroxidases (GPX1, GPX2, GPX3, GPX4, and GPX6)

Glutathione peroxidases (GPx) have a thioredoxin fold that is distinguishable from other selenoproteins, in which there is an alpha helix with a cysteine residue. This “Cys block” allows for interactions with thioredoxins. The selenocysteine residue is located on the N-terminus of this alpha helix. GPx proteins function as hydroperoxide and lipid-hydroperoxide oxidoreductases. GPX1 and GPX 4 are glutathione-dependent, whereas the others are not (Deponte, 2013).

During embryonic brain development, the guanine-rich sequence-binding factor 1 (Grsf1) up-regulates GPX4, and together they prevent developmental delays that are caused by apoptosis and lipid peroxidation (Ufer et al., 2008). One study found that a genetic knockout of GPX4 resulted in a similar developmental phenotype as that of a knockout for TRSP, the tRNA encoding Sec. Loss of GPX4 led to neurodegeneration and reduction of PV+ interneurons (Nahar et al., 2021), which are sensitive to oxidative stress (Wirth et al., 2010).

In adult brains, GPX4 is detected in the neurons of the cerebral cortex, hippocampus, and cerebellum. When GPX4 is removed from mice models, adults lost body weight and died within 2 weeks due to mitochondrial damage, decreased electron transport chain activity (Yoo et al., 2012). One study found that GPX4 was expressed in astrocytes within lesioned areas (Savaskan et al., 2007). The lack of GPX1 has also been implicated in a higher level of apoptosis (Crack et al., 2001; Flentjar et al., 2002) and larger lesion sizes (Klivenyi et al., 2000) when neurotoxic treatments are injected into brains. Further, a common genetic variant of GPX1 was found to be under transmitted from parents to children with autism spectrum disorder, suggesting a protective effect of a wild-type GPX1 (Ming et al., 2010). Together, these data suggest that GPX1 and GPX4 play an essential and protective role even in adulthood.

Ferroptosis is a recently identified type of programmed cell death that is differentiated from apoptosis in that it is associated with uncontrolled lipid peroxidation in cell membranes. The process is associated with oxidative stress in cancer and stroke, among other conditions. When selenium is injected into brains, GPX4 expression increases to avoid ferroptosis and stroke behaviors are circumvented (Alim et al., 2019). It has also been shown that Trx1 (a thioredoxin selenoprotein) regulates GPX4 expression to inhibit ferroptosis (Bai et al., 2021). The function of glutathione peroxidases may come into light with the discovery and investigation of ferroptosis.

### Thioredoxin reductases (Trx1, Trx2, and Trx3)

Thioredoxin reductases reduce protein disulfides through oxidoreductase function using NADPH as a substrate. In humans, they function as regulators of transcription factors, apoptosis, and immunomodulation (Arner and Holmgren, 2000). Thioredoxin reductases are also involved in the central nervous system and are well-established as neuroprotective factors (Hori et al., 1994; Stevanovic et al., 2020). Exome sequencing of an adolescent with neurodegenerative disorder identified a homozygous stop mutation in thioredoxin 2 (Trx2). Upon further examination using patient-derived fibroblasts, cells showed higher reactive oxygen species and impaired oxidative stress response (Holzerova et al., 2016). Further, mice who are born deficient in Trx1 developed severe ataxia and were smaller in size than controls, suggesting an impact on the cerebellum (Soerensen et al., 2008). In contrast, overexpression of Trx1 significantly decreased levels of reactive oxygen species and improved motor function (Liu et al., 2021), and may also protect from brain toxicity and addiction from drugs such as methamphetamine (Yang et al., 2020). The mechanism through which Trx1 exhibits antioxidant activity is through inhibiting intracellular ROS levels, DNA fragmentation, and regulating apoptosis (Yeo et al., 2021). Therefore, the thioredoxin reductases, like most other selenoproteins, play a neuroprotective role.

### Deiodinases (DIO1, DIO1, and DIO3)

Deiodinases have a conserved thioredoxin-fold domain consisting of beta-alpha-beta and beta-beta-alpha motifs (Bianco and da Conceicao, 2018). The Sec residue is presumed to be in the active site of the ER transmembrane protein (Bianco et al., 2002). The three deiodinases shared about 50% sequence similarity, and share a common function: DIO1 and DIO2 activate thyroid hormones T_3_ and T_4_, while DIO3 inactivates them (Bianco and da Conceicao, 2018).

One study examined DIO1 and DIO2 activity in brains of selenium-deficient rats. Brain DIO1 activity was unchanged by selenium deficiency. In contrast, brain DIO2 activity decreased in selenium-deficient rats. Both results suggest that deiodinase activity and, subsequently, T_3_ levels, are maintained and is the last to decrease in the brain over other organs when selenium deficient. Interestingly, markers of brain development also decreased when rats were deficient in selenium. This potentially suggests a relationship between deiodinases and brain development, though likely through thyroid hormone mechanisms (Mitchell et al., 1998).

### Selenoprotein P (SELENOP, SepP)

SELENOP is unique in that is has 10 Sec residues, as opposed to 1 sec residue in all other selenoproteins (Hill et al., 1993). Because of this, selenium associated with SELENOP accounts for 60% of total body selenium (Read et al., 1990). Seminal research in the field identified SELENOP as a transport carrier, with evidence of increased hepatic selenium (Schomburg et al., 2003) and decreased selenium in the testes, brain, and kidney in SELENOP-knockout mice (Hill et al., 2003). Further, lack of SELENOP led to increased urinary output of selenium, suggesting that SELENOP is involved in transporting selenium to target tissues (Burk et al., 2006).

SELENOP is expressed itself in many tissues, including the brain (Scharpf et al., 2007). It is known as a transporter of selenium from the liver to other tissues, and this process has recently been identified to occur through exosomes (Jin et al., 2020). There is competition between the brain and testes for selenium, and when SELENOP is deficient, severe neurodegeneration and neurological dysfunction occur unless castration occurs (Pitts et al., 2015), and in female models, this neurological impairment is milder (Kremer et al., 2019). When SELENOP is knocked out of a mouse model, irreversible neurological dysfunction such as inability to walk and stiff gait was noticed very soon after birth (Hill et al., 2004). Upon further examination of the hippocampus, synaptic transmission, short-term plasticity, and long-term potentiation were also adversely affected by SELENOP deficiency. This ultimately led to spatial learning differences when compared to controls (Peters et al., 2006). SELENOP’s hippocampal function may be mediated through Zinc homeostasis, demonstrated in an Alzheimer mouse model (Yue et al., 2020). A recent study also found that exercise-induced neurogenesis that occurs in the adult hippocampus is mediated by SELENOP (Leiter et al., 2022). Interestingly, children with intellectual disability have significantly lower serum levels of SELENOP (Gorlich et al., 2022), suggesting that the transport protein plays an essential role in proper brain development. Taken together, SELENOP’s Se transport function likely plays a significant role in the brain, particularly within the hippocampus.

### Selenoprotein R (SELENOR, SELR, and MsrB1)

SELENOR is a methionine sulfoxide reductase. The protein is conserved among the majority of living organisms. Unlike other selenoproteins, SELENOR is associated with zinc and it stereospecific (Kryukov et al., 2002). The selenocysteine residue within SELENOR seems to be integral to its antioxidant function in the cytoplasm and nucleus (Bar-Noy and Moskovitz, 2002; Kim and Gladyshev, 2004; Kim et al., 2006). While there has not been much published on SELENOR’s function in the brain, one study found that it is highly expressed within microglia and astrocytes in the central nervous system, relative to neurons (Shi et al., 2019). The loss of SELENOR led to astrocyte migration and astrogliosis in the hippocampus, which ultimately impacted spatial learning. While SELENOR-knockout mice swam as fast as the controls, it took a longer time for them to locate a hidden escape platform, and did not return to previously successful routes, indicating an impact on both spatial learning and memory. Further, the knockout mice exhibited long-term potentiation and long-term depression abnormalities as well (Shi et al., 2019). More work is needed to fully understand SELENOR’s function within the brain, though preliminary literature has identified an essential antioxidant function within the hippocampus.

### Selenoprotein T (SELENOT, SELT)

SELENOT is one of three selenoproteins which have a conserved Cys-X-X-Sec motif within a thioredoxin-like fold, which is characteristic of redox function (Dikiy et al., 2007). The presence of a hydrophobic transmembrane domain led to the discovery of SELENOT as integrated within the ER membrane. There, it closely resembles thioredoxin reductase activity, as it has been seen to reduce 5,5′-dithio-bis to 5-thio-2-nitrobenzoic acid in the presence of NADPH (Boukhzar et al., 2016).

There is an increased expression of SELENOT during embryogenesis, which quickly declines after birth. Specifically, SELENOT was found in the ER of immature neural cells from the forebrain, midbrain, and hindbrain of mouse embryos (Tanguy et al., 2011). In a study with SELENOT-knockout mice, littermate brains were 17% smaller in volume than those of wild-type mice (Castex et al., 2016). Upon further evaluation, the smaller brains of these littermates were likely due to an increase in caspase-3 activity by 53%, which was associated with a 46% increase in reactive oxidative species (ROS) levels. When mice are deficient in SELENOT, hyperactive behaviors are also noticed, such as higher mean velocity and lower immobile times (Castex et al., 2016). As a thioredoxin, SELENOT likely plays a neuroprotective role in redox homeostasis in neurons during brain ontogenesis.

### Selenoprotein W (SELENOW, SELW)

SELENOW contains a bound glutathione molecule at a cysteine residue in addition to the characteristic selenocysteine residue, both of which are essential for its antioxidant function. Along with SELENOT and SELENOH, SELENOW contains a conserved Cys-X-X-Sec motif and a thioredoxin-like fold, suggesting antioxidant properties. As predicted, SELENOW removes intracellular peroxide in a glutathione-dependent manner (Jeong et al., 2002). SELENOW interacts with protein 14–3-3 and this interaction increases in the presence of diamide or H_2_O_2_ (Jeon et al., 2016), suggesting a role in redox cell signaling. It plays a protective anti-inflammatory function across the body, including the heart (Liu et al., 2016) and immune system (Yu et al., 2015).

Using immunoblot analysis and *in situ* hybridization, SELENOW was identified at high levels in the brain and spinal cord of rat embryos and was restricted to the nervous system by fetal day 20 (E20). In addition, when SELENOW was placed on a human fetal brain cDNA library, it was identified to interact with FAM96B, which was verified by co-immunoprecipitation. FAM26B is known to modulate ferritin levels and genome integrity, both of which have been implicated in neurological disorders such as Alzheimer’s disease (Chen et al., 2019). These data implicate SELENOW with proper brain development.

By postnatal day 25 (P25), SELENOW is localized to the hippocampus, with moderately high levels in the cerebral cortex, dentate gyrus, and the cerebellum as well in rat newborns. The SELENOW levels in the hippocampus and cerebellum last through adulthood (Yeh et al., 1997; Raman et al., 2013). When sheep are either supplemented with or deprived of selenium, their brains maintained similar levels of SELENOW, suggesting the critical need for SELENOW homeostasis in the brain (Yeh et al., 1997). Therefore, SELENOW is an essential antioxidant for both brain development and function (Jeong et al., 2004). SELENOW has also been identified within synapses, along with several other proteins involved in selenoprotein synthesis (Raman et al., 2013). It has been seen in chicken neurons that SELENOW interacts with d-amino acid oxidase downstream of d-serine signaling to prevent oxidative stress-related neurotoxicity (Li et al., 2018).

### Selenoprotein I (SELENOI, EPT1)

SELENOI, also known as ethanolamine phosphotransferase 1, is a transferase enzyme involved in the synthesis of phosphatidylethanolamine (a cellular phospholipid). It contains a CDP-alcohol phosphatidyltransferase motif, common in phospholipid synthases. Unique to this selenoprotein, it seems that SELENOI is not directly involved in antioxidant activity, but rather the products of its function (i.e., phosphatidylethanolamine) play a role in defending against oxidative stress, along with its role within the lipid bilayer of cellular organelle membranes (Horibata et al., 2018). SELENOI is expressed in multiple tissues, but within the brain it is particularly expressed within the cerebellum (Horibata and Hirabayashi, 2007). An exon-skipping mutation in SELENOI was discovered in a patient with hereditary spastic paraplegia, deafness, and blindness, with brain atrophy affecting the cerebellum and midbrain (Horibata et al., 2018). Little is known regarding SELENOI in the brain, and further work is recommended to understand mechanisms behind SELENOI function.

## Oxidative stress in ASD

As discussed, most selenoproteins function as protection from oxidative stress, particularly within the hippocampus and cerebellum of the brain. ASD etiology is established as linked with oxidative stress in the brain (Raymond et al., 2014; Bjorklund et al., 2018; Hu et al., 2020; Manivasagam et al., 2020; Pangrazzi et al., 2020). Sources of oxidative stress during neurodevelopment may include maternal immune activation and environmental toxins, both of which have been linked to ASD. However, the compensation of partially functioning proteins such as SHANK3, FMRP, or CHD8 during neural development would require increased metabolic activity and, subsequently, more oxidative stress.

Studies examining biomarker levels in children with ASD found a significantly higher level of oxidative stress markers and plasma glutamate in comparison to neurotypical controls (Adams et al., 2011). The leading explanation for the high levels of oxidative stress biomarkers is an imbalance in selenoprotein antioxidant defense (Raymond et al., 2014), such as glutathione. One meta-analysis found decreased blood levels of glutathione and glutathione peroxidases in ASD patients relative to controls (Frustaci et al., 2012), along with three independent studies published after the meta-analysis (Chauhan et al., 2012; Rose et al., 2012; Gu et al., 2013). This glutathione expression and activity imbalance seem to be within the cerebellum and temporal cortex. Aside from glutathione, other antioxidants such as ceruloplasmin and transferrin have been seen at lower levels in serum of ASD patients (Chauhan et al., 2004); interestingly, while these are not selenoproteins, ceruloplasmin and transferrin are associated with transport of copper and iron, respectively. Of note, some discuss high levels of oxidative stress markers as an independent factor of ASD etiology, rather than a result of antioxidant imbalance (Chauhan and Chauhan, 2006).

The increased oxidative stress in the brain may be due to impairments in the blood–brain barrier (BBB): one study found that ASD patients have lower levels of endothelial adhesion molecules, which suggest blockage of peripheral leukocytes into the central nervous system (Onore et al., 2012). Another group identified alterations in BBB integrity in post-mortem brain tissues of ASD patients, indicated by varied expression levels of tight junction and similar BBB molecules (Fiorentino et al., 2016).

## Selenium and selenoproteins in autism spectrum disorder

Prior to our study described above, it has been suggested that dyshomeostasis in selenium may be associated with ASD incidence (Raymond et al., 2014). Since our study, others have also reported an association between ASD and low plasma Se levels (Wu et al., 2022; Zhang et al., 2022). However, as a whole, the literature on this subject has been equivocal. In a literature review of 10 studies comparing hair trace element levels in ASD and controls, four of them found a significant difference in Selenium levels. Two found a significant increase in Selenium levels in children with ASD, and two found a significant decrease (Tinkov et al., 2019). Another meta-analysis found no significant differences in mean hair or erythrocyte selenium concentrations among 12 studies (Saghazadeh et al., 2017). A retrospective study of prenatal levels of trace elements in Norwegian mothers found no association between Se levels and risk for Autism in pregnancy (Skogheim et al., 2021) while a prospective study in the United States found an associated between elevated maternal Se levels and increased ASD risk (Lee et al., 2021). There are several possible factors that account for the variability of these studies. The first is variability of measurement. Older studies used a variety of techniques to measure Se concentration, rather than the inductively coupled plasma mass spectrometry (ICP-MS) that has become standard over the past few years. Second, the source material in these studies varied significantly. In both ASD and control populations, the variability of Se in hair and nails was greater than in serum (Mehta et al., 2021). Finally, there is evidence that normal ranges of Se change depending on age and gender (e). Many previous studies recruited children with ASD from ages 2–18, which may have resulted in non-significant findings.

**Figure 2:**
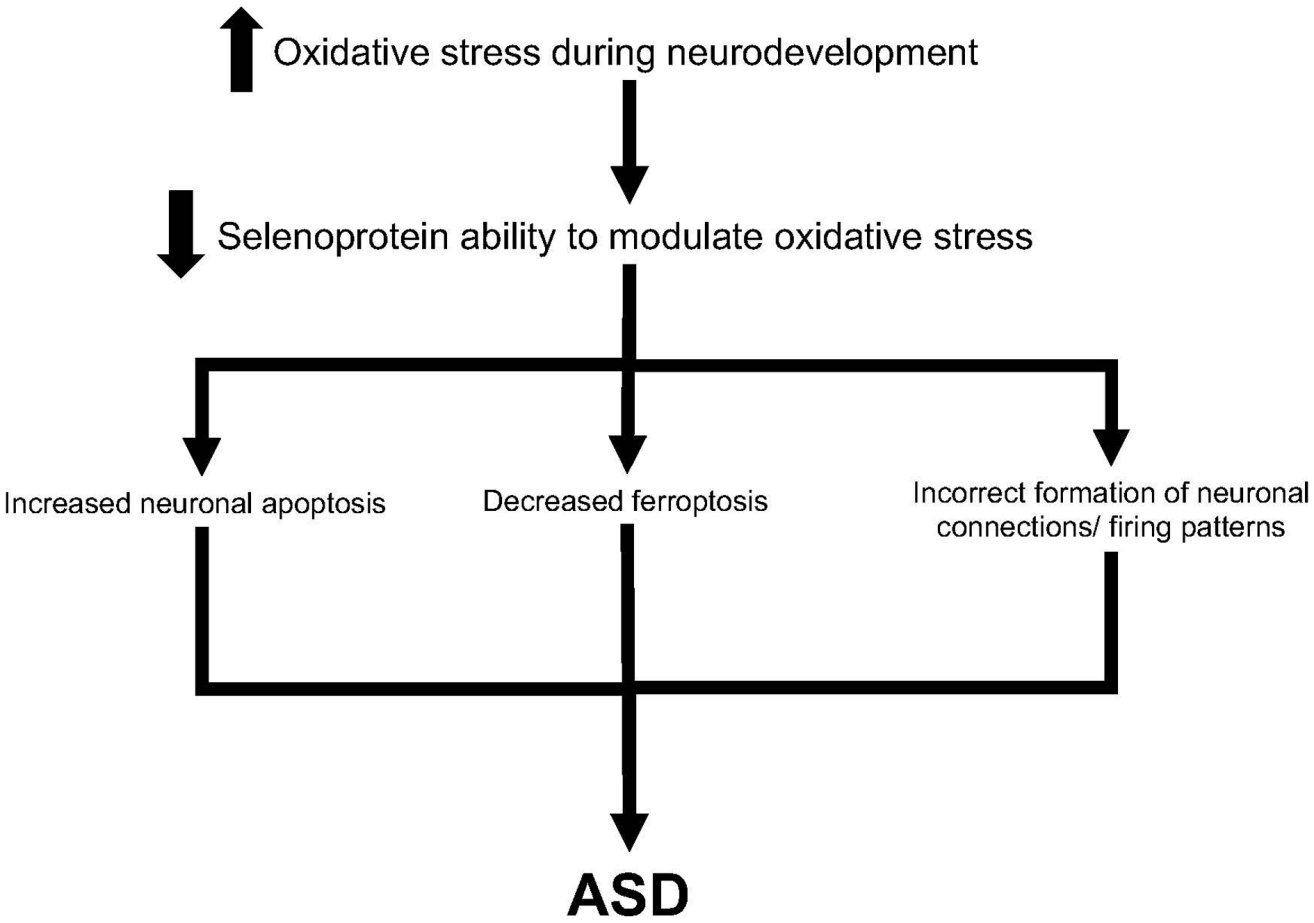
Hypothesis for ASD development through increased oxidative stress and selenoproteins. Increased oxidative stress may be through maternal immune activation, environmental factors, or compensation from partially-functioning proteins such as SHANK3. This may lead to decreased ability of selenoproteins such as glutathiones to modulate oxidative stress. As such, increased neuronal apoptosis, decreased ferroptosis, and incorrect formation of neuronal connections may lead to eventual development of ASD.

We propose several hypotheses for the etiology of ASD related to selenoproteins (Figure 2). Increased oxidative stress during neurodevelopment leads to a lessened ability for selenoproteins to function efficiently. Thus, oxidative stress may lead to increased neuronal apoptosis, as seen in many studies investigating glutathione peroxidases 1 and 4 (Crack et al., 2001; Flentjar et al., 2002; Ufer et al., 2008). Second, ferroptosis may be decreased in oxidative stress conditions. It has been shown that TRX1 and GPX4 work together to modulate ferroptosis (Bai et al., 2021). Finally, oxidative stress may lead to incorrect formation of neuronal connections and/or firing patterns during neurodevelopment. Raymond et al. hypothesized that the antioxidant function of glutathione and other selenoproteins affect the epigenetic regulation of gene expression (Raymond et al., 2014), thus altered oxidative stress levels in the brain may lead to abnormal neuronal connections. Taken together, there are several plausible hypotheses for the etiology of ASD through oxidative stress mechanisms and selenoproteins function.

## Selenium supplementation

We attempted to establish a pattern of selenium formulation used as a dietary supplement in clinical trials using information available at the www.clinicaltrials.gov. The keyword “Selenium” was searched in www.clinicaltrials.gov website, which resulted in 381 hits. Of those, only 222 studies were completed. To which, only 69 studies were conducted on children under age 17 and only one in ASD. The alone study was conducted in Indonesia to study the “Effect of High Selenium Functional Food and Selenium Supplement” (NCT05218577). Interestingly, this study used beef liver as a source of selenium supplement. However, when we searched with condition keyword as “Autism” and others as “selenium” we found total two studies focusing on Autism including the first one conducted in Indonesia (NCT05218577). The second study was performed at the Arkansas Children’s Hospital Research Institute (NCT00572741), where investigators studied treatment of oxidative stress and the metabolic pathology of autism with multi-vitamins and multi minerals containing 50 mcg of Selenium.

We thought to perform additional search for “Selenium supplementation” alone irrespective of its application in different disease pathology. We got 95 hits. Of those only 63 were completed. When we attempted to establish a selenium formulation pattern in these studies, we found variation in type of selenium used in these clinical trials. The major forms of Selenium used in these studies were, Selenium-yeast, sodium selenite (selenase) and selenomethionine. However, in many studies with limited information available on the clinicaltrials.gov website, it was not clear which chemical form of the selenium was used, especially when selenium was part of multi nutrients containing various vitamins, minerals including selenium, but no specific chemical form of selenium was mentioned making it even harder to establish preferred chemical form of Selenium. In our opinion, every chemical form of selenium has its own advantages and disadvantages in terms of bioavailability, toxicity, suitability of absorption, ease of synthesis and formulation.

**Figure 3:**
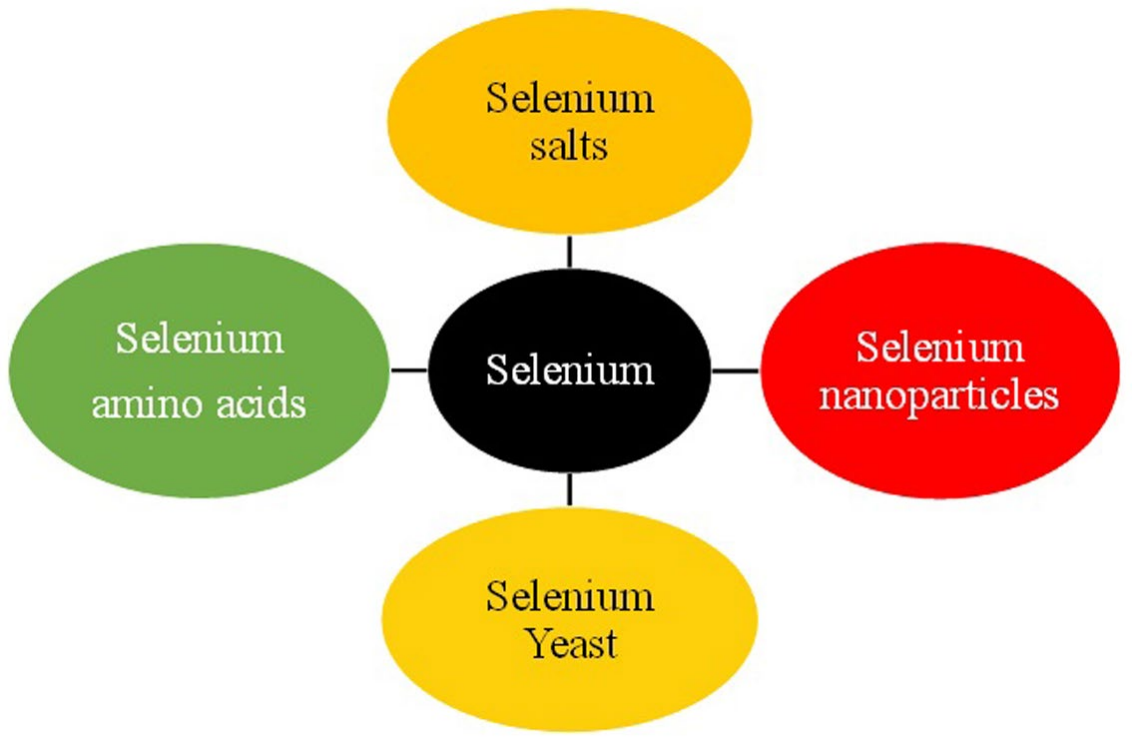
Possible ways of Selenium supplementation.

Based on the chemical composition and formulation, selenium supplementation can be categorized in following four categories, (i) Selenium enriched yeast, (ii) Selenium containing amino acids (iii) Selenium salts (iv) Selenium nanoparticles (Figure 3).

1. *Selenium enriched yeast*: Selenium enriched yeast is simply known as a selenium yeast and produced by culturing *Saccharomyces cerevisiae* in a selenium rich media. *Saccharomyces cerevisiae* commonly known as a brewer’s yeast, which produces selenomethionine in presence of selenium along with normal methionine due to the similar chemical characteristics of selenium as of sulfur. Irrespective of amount of selenium content in a culture media, complete replacement of methionine by selenomethionine has not been reported. The highest quantity of selenomethionine produced by a single yeast cell is 3,000 ppm (Schrauzer, 2006). Various safety concerns have been raised to selenium yeast as a dietary supplement due to variable quantities of selenomethionine present in selenium yeast and there was no rigorous quality control check for the presence of other contaminations. However, later some quality control parameters were placed to ensure the safety of selenomethionine produced from the *Saccharomyces cerevisiae as a* selenium yeast including limits on moisture content in the yeast, limits on allowed lead and arsenic presence, limits on allowed live bacterial and mold counts (Schrauzer, 2006). Moreover, selenium yeast is used as feed additives to supplement selenium.
2. *Selenium containing amino acids*: Two major source of selenium in the form of amino acid are selenomethionine and selenocysteine. Both these amino acids can be obtained either by biochemical synthesis or by chemical conversion of Se into selenomethionine (yeast) and selenocysteine (plants) (Whanger, 2002; Rahmanto and Davies, 2012; Schiavon et al., 2020). Various chemical reactions have been developed to synthesize selenomethionine (Krief and Trabelsi, 1989; Iwaoka et al., 2008) and selenocysteine (Iwaoka et al., 2008) as a large-scale source of synthetic Se containing amino acid. Selenocysteine as an United States Pharmacopeia (USP) grade reference standard is also commercially available.
3. *Selenium salts*: Another source of selenium is from plant-based diet, where plants accumulate selenium in the form of inorganic compounds like selenates, Na_2_SeO_3_ (Se^+4^) and Na_2_SeO_4_ (Se^+6^) and eventually convert selenium into organic selenium like selenomethionine, selenocysteine or other selenium containing amino acids (Kieliszek, 2019; Schiavon et al., 2020). The biosynthesis of selenocysteine has been reported to show a stepwise conversion of inorganic selenium to organic selenium (Schiavon et al., 2020).
4. *Selenium nanoparticles*: To reduce the toxic effect of inorganic selenium and to enhance the bioavailability of selenium, nanoparticle-based formulation of selenium has been explored, where selenium is encapsulated in the core of the nanoparticle assembly and used as delivery vehicle. Based on the published preclinical studies, nanoparticle-based selenium formulation has shown enhanced bioavailability, control release and lower toxicity than other selenium formulations (Constantinescu-Aruxandei et al., 2018; Hosnedlova et al., 2018; Bisht et al., 2022).

## Perspectives and conclusion

Selenium and selenoproteins are involved in healthy brain function and development, primarily through their antioxidant functions. Oxidative stress is associated with ASD; studies show a correlation between increased oxidative stress biomarkers and presence of ASD. Many have also shown a decrease in selenoproteins such as glutathiones in ASD patients, suggesting that selenoprotein dyshomeostasis may in fact play a role in ASD etiology. However, current literature studying selenium dyshomeostasis and ASD are variable in nature and thus inconclusive. Further, selenium supplementation trials (whether with ASD cohorts or others) lack clear definitions of the type of selenium used for supplementation. Larger and better-defined trials are needed to better understand the role of selenium and selenoproteins in the etiology of ASD, as well as the ideal type of selenium needed for supplementation trials.

## Author contributions

SB, SM, and MP contributed to conception and design of the review. SB wrote the first draft of the manuscript. SM and MP wrote sections of the manuscript. All authors contributed to manuscript revision, read, and approved the submitted version.

## Conflict of interest

The authors declare that the research was conducted in the absence of any commercial or financial relationships that could be construed as a potential conflict of interest.

## Publisher’s note

All claims expressed in this article are solely those of the authors and do not necessarily represent those of their affiliated organizations, or those of the publisher, the editors and the reviewers. Any product that may be evaluated in this article, or claim that may be made by its manufacturer, is not guaranteed or endorsed by the publisher.
